# Wound Healing in Butterfly Pupal Wing Tissues: Real-Time In Vivo Imaging of Long-Range Cell Migration, Cluster Formation, and Calcium Oscillations

**DOI:** 10.3390/insects16020124

**Published:** 2025-01-27

**Authors:** Shuka Nagai, Joji M. Otaki

**Affiliations:** 1 The BCPH Unit of Molecular Physiology, Department of Chemistry, Biology and Marine Science, Faculty of Science, University of the Ryukyus, Nishihara, Okinawa 903-0213, Japan; 2 Department of Molecular Neuroscience, Graduate School of Frontier Biosciences, Osaka University, Suita, Osaka 565-0871, Japan

**Keywords:** butterfly wing, calcium oscillation, color pattern formation, eyespot, real-time in vivo imaging, scale development, wound healing

## Abstract

Insects can repair wounds and regenerate body parts in response to physical damage. Wound healing in butterfly pupal wing tissues is developmentally interesting because ectopic color patterns develop during healing, suggesting that normal and damage-induced color patterns may use similar mechanisms. Here we physiologically investigated wound healing and ectopic color pattern formation in butterfly pupal wing tissues using the blue pansy butterfly *Junonia orithya*. In response to physical puncture damage, various ectopic color patterns are formed around the damage site. After the wounding operation, we observed hemocytes migrating over long distances along the wing veins (lacunae) toward the damage site, where hemocytes and epidermal cells formed cellular clusters. Calcium oscillations were observed in cells at and near the damage site. Calcium oscillations were transiently affected by ruthenium red, an inhibitor of calcium transporters and channels, and ruthenium red caused various abnormalities in the scales of adult wings. These results suggest that cell migration, cluster formation, and calcium oscillations play important roles in wound healing and scale development at and near the damage site. Ectopic color patterns may develop in response to local calcium oscillations as a consequence of the evolutionary co-option of the healing process for normal development.

## Simple Summary

This study focused on wound healing and accompanying ectopic color pattern formation in butterfly pupal wing tissues using the blue pansy butterfly *Junonia orithya*. In response to physical damage, various ectopic color patterns formed around the damage site in adult wings. After the wounding operation, we observed hemocytes migrating over long distances toward the damage site, where hemocytes and epidermal cells formed cellular clusters. Calcium oscillations were observed in cells at and near the damage site. Calcium oscillations were transiently affected by ruthenium red, an inhibitor of calcium transporters and channels, and ruthenium red caused various abnormalities in the scales of adult wings. These results suggest that cell migration, cluster formation, and calcium oscillations play important roles in wound healing and scale development at and near the damage site.

## 1. Introduction

Wound healing and regeneration are important physiological processes for the survival of animals against accidental mechanical damage to the body in most animal phyla, including Arthropoda [1,2,3]. In insects, the outermost barrier is cuticular exoskeletons, which are lined with epidermal (epithelial) cells [4]. When these integumentary cuticles and cells are physically damaged, insects can repair wounds and often regenerate lost body parts. To achieve wound repair, insects must restore cuticular structures with sufficient mechanical rigidity via targeted cuticle deposition [5,6]. In the research field of wound healing and regeneration, *Drosophila* larval imaginal disc regeneration and embryonic dorsal closure during development have been studied as model systems to understand the cellular and molecular mechanisms of wound healing and regeneration, which may be applicable not only to invertebrates but also to vertebrates [7,8,9,10,11,12,13].

In *Drosophila* systems, actin networks play important roles in the process of wound healing [14]. Actomyosin filaments assemble at the wound edge, which is called actomyosin flow, during cytoskeletal remodeling [15], and the contraction of an actin purse string closes the wound [10,16,17]. Actomyosin remodeling requires polarized E-cadherin endocytosis [18]. Calcium signals play an important role in these processes [15]. For example, annexins are rapidly recruited to the wound edge in response to calcium signals, leading to the formation of actomyosin filaments [19]. Indeed, calcium signals are likely to be master regulators of the wound-healing process in *Drosophila* pupal wing tissues [20]. In response to mechanical damage, slow, long-range intercellular calcium waves occur, which are mediated by the IP_3_ receptor (and hence via IP_3_), a calcium pump called SERCA (sarcoendoplasmic reticulum Ca^2+^-ATPase), and gap junctions in the *Drosophila* wing imaginal disc [21,22]. In other words, calcium and/or IP_3_ travel intercellularly through the gap junctions. According to a mathematical simulation study, intercellular transfer of IP_3_ is required for long-range calcium signals around the wound [23]. During wound healing, mitochondrial fusion and/or fission may regulate calcium signals in *Drosophila* [24]. An important additional point is that mechanical forces should be provided to repair wounds physically [25]. In the *Drosophila* wing imaginal disc, spatiotemporal patterns of intercellular calcium signals appear to reflect cell shape anisotropy and may convey mechanical information to cells [26]. Calcium signals activate NADPH oxidase DUOX (a dual NADPH oxidase) for the production of hydrogen peroxide, which may act as an attractant for immune cells [27].

Wound healing may recapitulate the normal process of morphogenesis and vice versa; in this sense, *Drosophila* dorsal closure has been studied intensively [28]. Importantly, dorsal closure in *Drosophila* requires not only cell migration but also mechanical forces from purse string structures and other sources [29,30]. There is no doubt that mechanical forces are crucial for realizing morphogenesis and wound healing because they are mechanical processes involved in cellular activities [31]. To be sure, many molecules are involved in wound healing [32,33] and dorsal closure [34], including extracellular matrix molecules [35]. Many molecules, such as cytokines, are involved in the recruitment of hemocytes in *Drosophila* [36]. Mechanical forces and cellular and molecular dynamics should be coordinated, as modeled in *Drosophila* dorsal closure [37].

In concert with these signaling and remodeling activities of the epidermis, physical damage causes innate immune responses [38,39,40]. In *Drosophila*, most hemocytes are plasmatocytes, which have professional phagocytic functions similar to those of mammalian macrophages to remove cellular debris in concert with other processes of wound healing and regeneration [4,38,39,40]. In *Drosophila*, another type of hemocyte, crystal cells, are involved in melaninization via phenoloxidase, which is a part of the innate immune system [4,38,39,40,41]. Melanogenesis via phenoloxidase plays various roles in insect physiology, including wound healing, sclerotization, coloration, and immunity [42,43]. Similarly, DOPA decarboxylase, which produces dopamine and serotonin, plays various roles in insect physiology, including wound healing, sclerotization, coloration, and immunity [44]. In one of the lepidopteran species, the cabbage white butterfly *Pieris rapae*, three types of hemocytes, namely, prohemocytes, plasmatocytes, and granulocytes, have been reported to constitute the major hemocytes [45,46].

In the pupal wing epidermal (epithelial) tissues of butterflies, puncture damage is repaired quickly, and repaired adult wings can be observed after eclosion [47]. Intriguingly, repaired damage sites often accompany eyespot-like ectopic color patterns [47,48,49,50,51]. Calcium oscillations and propagating waves are induced at the damage site but are also observed spontaneously from the prospective eyespot focus (i.e., the eyespot organizer) [52]. Moreover, the prospective eyespot focus expresses many kinds of genes involved in wound healing and calcium signaling [53]. Hence, it is understandable that normal color pattern formation during development and ectopic color pattern formation after wound healing are mechanistically similar in butterflies. Ectopic color patterns and calcium signals are induced by puncture damage, not only in butterflies but also in fish [54], suggesting that butterfly and fish systems share a common mechanism of wound healing and color pattern development to some extent. Although the cellular and molecular mechanisms of color pattern development in butterfly wings have been studied intensively using several approaches, including comparative morphological approach [48,55,56,57,58,59,60,61,62,63,64,65,66,67,68,69,70,71], surgical and physical approach [72,73,74,75,76], physiological approach [77,78,79,80], bioimaging approach [81,82,83,84,85,86], and genetic and molecular biological approach [87,88,89,90,91,92,93,94,95,96,97,98,99,100,101,102,103,104,105,106,107], the wound-healing process itself has not been studied at the cellular level in butterflies.

In the present study, we first demonstrated that puncture damage to pupal wing epidermal tissues induced wing repair and ectopic color patterns in our experimental species, *Junonia orithya*. Damage-induced ectopic color patterns in this species have already been demonstrated [51]; however, we first confirmed the ectopic color patterns here before proceeding with further experiments. Following this confirmation, we focused on cellular dynamics and calcium oscillations in pupal wing tissues after puncture damage using a bioimaging technique. The butterfly pupal wing system mainly contains only two types of cells: epidermal (epithelial) cells that constitute the wing epidermis and free-moving hemocytes, and we differentially stained these two types of cells. We further showed that a general calcium inhibitor, ruthenium red, affected wing scale development, although the effect of thapsigargin, an inhibitor of Ca^2+^-ATPase (SERCA), on wing development in *J. orithya* has already been documented [52,108]. We then examined the effects of calcium inhibition by ruthenium red on damage repair and ectopic color patterns in the present study. The present results provide fundamental information on the process of wound healing in terms of cellular migration and calcium signaling in butterfly wings.

## 2. Materials and Methods

### 2.1. Butterflies

The blue pansy butterfly *Junonia orithya* (Linnaeus, 1758) was used throughout this study. We focused on the dorsal side of the hindwing. Female butterflies were captured in the central and southern regions of Okinawa-jima Island, Japan, mainly on the Nishihara Campus of the University of the Ryukyus. The females were confined to a glass tank to obtain eggs. Larvae were reared mainly with the natural host plant *Plantago asiatica*.

### 2.2. Staining and Wounding Operations

In this study, the operations were performed on the dorsal hindwing. Within one hour of pupation, the forewing tissue was lifted with forceps. To stain epidermal cells, fluorescent dyes or indicators (20 μL) were placed on the exposed dorsal hindwing tissue, and the forewing tissue was placed back to the original position, which is called the sandwich method. The treated pupae were left intact for one hour, after which the forewing tissue was lifted again, and then the surface of the hindwing tissue was washed with Insect Ringer solution. After this staining procedure (approximately 2 h postpupation), a puncture wound was made manually using a Shiga Konchu Insect Pin (size no. 06; 0.65 mm in diameter) (Tokyo, Japan) directly on the hindwing tissue at a depth of approximately 1.0–2.0 mm. This wounding operation was performed to pierce the hindwing tissue without losing the hemolymph from the wound site. The pin was moved up and down manually to ensure the wounding process. We obtained a single hole in most cases but occasionally obtained multiple holes due to the inaccuracy of the pin movement. We took advantage of this variability in this study. A wound was made in the medial background area of the hindwing between the anterior and posterior eyespots. The hindwing tissue surface was subsequently washed again with Insect Ringer solution, directly placed on a thin glass plate, and then optically observed. Alternatively, after the staining procedure via the sandwich method to stain epidermal cells, an injection (2 μL per pupa, unless otherwise indicated) was made at the pupal abdomen to stain hemocytes using an Ito microsyringe (Fuji, Shizuoka, Japan). These simultaneous treatments with the sandwich and injection methods were performed to differentially stain these two cell types. When the wounding operation was performed without the forewing lift, a puncture wound was placed in the medial background area of the hindwing between the anterior and posterior eyespots at a depth of approximately 2.0–2.5 mm from the pupal surface.

### 2.3. Chemicals

The following fluorescent dyes (with final concentrations) were used for cellular staining: MitoRed for mitochondria (Dojindo Laboratories, Kumomato, Japan) (11.1 μM for sandwich and 250 or 333 μM for injection), Hoechst 33342 for nuclei (Dojindo) (320 μM for sandwich), BODIPY FL C_5_-ceramide complexed to BSA for membranous structures (Thermo Fisher Scientific, Tokyo, Japan) (27.8 or 33.3 μM for sandwich), SYBR Green I (Takara Bio, Kusatsu, Shiga, Japan) (4.44 × 10^6^ times dilution of the original solution), and fluorescent brightener 28 disodium salt solution (FB28) for chitin (Sigma Aldrich, St. Louis, MO, USA) (4.17% for sandwich). FB28 has been demonstrated to be useful for observing chitin dynamics in butterfly pupal wing tissues in vivo [80,86]. These fluorescent dyes were diluted with DMSO (dimethyl sulfoxide) and/or Insect Ringer solution.

To image hemocytes, we mainly used MitoRed via the injection method. There are three reasons for using MitoRed instead of LysoTracker Red, or something similar that is more specific to a certain type of hemocyte. First, this insect system is very simple, mainly containing only two types of cells: epidermal (epithelial) cells and hemocytes. Accordingly, most, if not all, free-moving cells in the hemolymph are considered hemocytes [83,84,85,109]. Second, LysoTracker Red can efficiently label hemocytes with lysosomes [84]; however, it is difficult to label other types of hemocytes without lysosomes. We did not intend to differentiate subtypes of hemocytes in this study. Third, in contrast to LysoTracker Red, most, if not all, hemocytes appear to be highly sensitive to MitoRed. Fortunately, all types of hemocytes seem to have many active mitochondria, which enables us to detect all types of hemocytes, regardless of the presence of lysosomes. This high level of staining is important for time-lapse imaging for hours, in which bleaching of fluorescent dyes is the major obstacle. Therefore, it is important to use a generic dye, MitoRed, which can stain many types of free-moving cells in the hemolymph. To stain epidermal cells, we mainly used BODIPY via the sandwich method.

For calcium imaging, CalBryte 590 AM (AAT Bioquest, Pleasanton, CA, USA) (20 mM for sandwich) was used. CalBryte 590 AM was loaded onto the hindwing tissues via the sandwich method, as described above. When the tissue was also stained with BODIPY, CalBryte was first loaded, and then BODIPY was loaded. To block calcium signals, ruthenium red (Cayman Chemicals, Ann Arbor, MI, USA) (10 mM for injection) was dissolved in ultrapure deionized water and used for injections. We injected 2 or 3 μL of ruthenium red solution using an Ito microsyringe. We did not perform injections of water as a negative control in this study because it is known that the injection of water does not induce any effect on wing color patterns [77].

### 2.4. Imaging

For general cellular imaging and calcium imaging, we used a Nikon A1 real-time confocal laser microscope operated by NIS ELEMENTS AR 4.20.00 (Nikon, Tokyo, Japan). Excitation was performed at 405 nm, 488 nm, and 561 nm, and fluorescent signals were obtained at 425–475 nm, 500–550 nm, and 570–620 nm with a laser HIV 50–150 and a gain of 0.1–5.0. For calcium imaging, another Nikon A1 real-time confocal laser microscope operated by AQUACOSMOS 2.6 (Hamamatsu Photonics, Hamamatsu, Shizuoka, Japan) was also used. Excitation was performed at 561 nm, and fluorescent signals were obtained at 570–620 nm with a laser gain of 255. Time-lapse imaging of cellular dynamics started approximately 5–15 min after the damage treatment; during this period, the treated pupa was set at the microscope, and optical cross-section images were often taken before the start of time-lapse imaging. Once initiated, time-lapse images were obtained every 10 min for 2–3 h. For calcium imaging using CalBryte 590 AM, time-lapse images were obtained every 10 s for 1–2 h immediately after damage (within 5 min postwounding). We focused on changes in the fluorescence intensity in the regions of interest (ROIs), although the absolute fluorescence intensity varied from individual to individual. When an injection of ruthenium red was performed during calcium imaging, the image recording was continued, but images from this period (18–24 s) were discarded owing to the potential dislocation of the object. We avoided the potential dislocation of the object as much as possible; however, this possibility cannot be excluded. We also used a Keyence Digital Microscope VHX-7000 (Osaka, Japan) to observe scales at the damage sites on the adult butterfly wings. The cell size in micrometers squared was measured using ImageJ 1.54g (National Institute of Health, Bethesda, MD, USA), assuming that the cells were either enclosed with BODIPY or filled with MitoRed.

### 2.5. Statistical Analyses

After the cell sizes were determined, the means and standard deviations were calculated. To compare cell sizes between the two populations, a two-sided unpaired Student’s *t*-test was performed, and the results were visualized in box plots using Microsoft Excel (Microsoft Office 365). The number of individuals with scars or holes at the damage site was counted and tested for statistical significance with Fisher’s exact test (two-sided) using JSTAT 16.1 (Yokohama, Japan). The repair state (degree of healing) for each damage hole was scored as 1 (no coverage), 2 (central coverage), 3 (full coverage), or 4 (full coverage with epidermal central coverage) based on the final images of the damage hole. Pearson’s correlation coefficient *r* and its associated *p*-value between size and healing score were obtained using JSTAT 16.1.

## 3. Results

### 3.1. Damage-Induced Wing Repair and Ectopic Color Patterns

**Figure 1:**
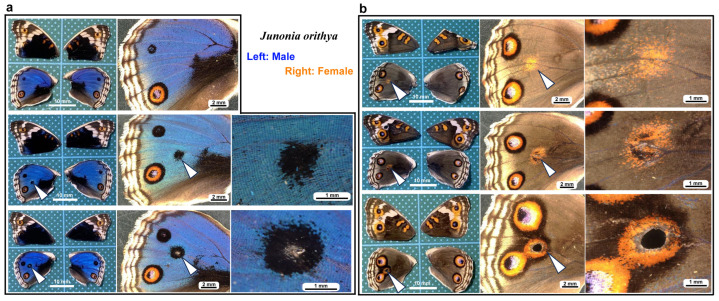
Damage-induced wing repair and ectopic color patterns. The panels in a single row represent the images from the same individual. Left wings were wounded, and the contralateral right wings served as controls. The arrowheads indicate the damage sites and the damage-induced ectopic color patterns. (**a**) Male individuals treated with a puncture wound. The bottom individual has a scar at the center of damage. (**b**) Female individuals treated with a puncture wound. The middle individual has a scar at the center of damage. The bottom individual has a hole (seen as a black area) at the center of damage.

We first tested whether puncture wounds in the hindwing induced repair and ectopic color patterns. Among the treated individuals (*n* = 30), various degrees of wing repair and ectopic color patterns were observed (Figure 1). In terms of ectopic color patterns, with the exception of one male individual (Figure 1a, top), all individuals presented damage-induced ectopic color patterns. In males, a black spot was often induced (Figure 1a, middle). An induced black spot was sometimes associated with a scar (an area without scales) at the center (Figure 1a, bottom). The induced black spot associated with a scar was larger than the induced black spot without a scar, suggesting that the induced black spot size was proportional to the severity of damage. In females, an orange spot was often induced (Figure 1b, top), and when a scar or hole was present, the induced orange spot was associated with an induced black core area (Figure 1b, middle) and/or an induced outer black ring (Figure 1b, bottom), forming an eyespot-like structure, suggesting that the severity of damage is proportional to the size and elaboration of induced color patterns. The ectopic eyespot-like structures fused with the normal eyespots, suggesting that their mechanisms of formation are fundamentally similar. In terms of wing repair, only four individuals presented with a scar without scales at the center of the damage (Figure 1a, bottom) or had a physical hole at the center (Figure 1b, middle). The other 26 individuals did not show any scars or holes, demonstrating efficient wound healing in butterfly pupal wing tissues. The unsealed hole was large (more than 1 mm in diameter), suggesting that the damage size was too large to completely seal the hole in this case. These results demonstrated that the puncture wound was efficiently repaired in most cases, but that the damage hole could not be filled when it was too large. These results also demonstrated that various ectopic color patterns were produced during wound healing, likely in proportion to the severity of damage.

### 3.2. Cellular Characterization at the Damage Site

To understand the cellular dynamics of the damage site, we first observed the wing area in an untreated individual corresponding to the damage site of a treated individual using a staining procedure that was applied to the treated wings; epidermal cells and other cells near or in the epidermal sheet were stained with BODIPY (green) for membranous structures and Hoechst 33342 (blue) for nuclei via the sandwich method, and hemocytes in hemolymph were stained with MitoRed (red) for mitochondria via the injection method. This differential staining method was designed to roughly differentiate epidermal cells and hemocytes in our system, which is mainly composed of these two cell types. Because these fluorescent dyes are generic, this method was not perfect, but we did not intend to identify the subtypes of these cell types. Importantly, MitoRed stained any free-moving hemocytes well (see below), which was suitable especially for long-term observations without bleaching.

**Figure 2:**
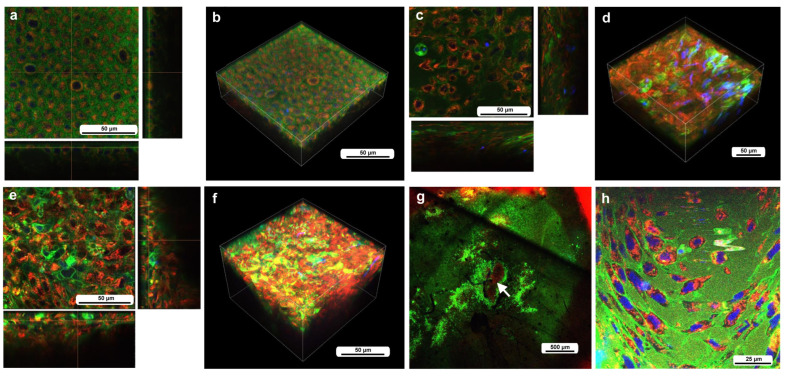
The cells at the nondamaged and damaged sites 5–6 h postwounding. These cells were stained with BODIPY, Hoechst, and MitoRed, although Hoechst staining was not very successful. The right and bottom rectangular panels associated with the main panel show optical cross sections along the *z*-axis. (**a**) Nondamaged site. (**b**) Three-dimensional reconstruction of (**a**). See Supplementary Video S1 for further details. (**c**) Damage site of an individual. (**d**) Three-dimensional reconstruction of (**c**). See Supplementary Video S2 for further details. (**e**) Damage site of another individual. (**f**) Three-dimensional reconstruction of (**e**). See Supplementary Video S3 for further details. (**g**) Damage site of yet another individual at low magnification (arrow). (**h**) High-magnification image of (**g**).

We first observed the cells without wounding at approximately 7–8 h postpupation. Although Hoechst (blue) did not work well, epidermal cells were stained with BODIPY (green), and their mitochondria were lightly stained with MitoRed (red) (Figure 2a,b), suggesting that the differential staining between epidermal (and associated) cells present on the surface and hemocytes present in the hemolymph was partially (although not completely) successful. Epidaermal cells were placed tightly, and the epidermal layer was one-cell thick (Figure 2a,b). Clearly, large and small cells were present. The small cells seemed to be stained with MitoRed more than the large cells. The large and small cells are likely prospective scale/socket cells and general epidermal cells, respectively.

We then observed the cells at the damage site in an individual with the wounding operation at approximately 5–6 h postwounding (7–8 h postpupation), assuming that the damage site was repaired at least partially by that time. In contrast to the previous individual without wounding, the cells were irregular in shape and disordered in position (Figure 2c,d). The cells also seemed to be larger in size. The tissue was much thicker than the untreated tissue, forming cellular clusters instead of a cellular monolayer. Importantly, the cluster was composed of two types of cells: one stained mainly with MitoRed (red) and the other stained mainly with BODIPY (green). The red cells were likely hemocytes, and the green cells were likely epidermal cells. Both red and green cells were flat, but the latter were located relatively near the apical surface. The damage site of the second individual treated in the same way showed similar results (Figure 2e,f). Again, the green cells were located relatively near the apical surface. The damage site of the third individual did not show cellular clusters, probably because the wound-healing process was halted earlier in this individual. This damage site seemed to be loosely covered by MitoRed-positive cells, around which BODIPY-positive cells were present (Figure 2g). In the high-magnification image, some of these MitoRed-positive cells at the damage site were stained simultaneously with BODIPY and Hoechst (Figure 2h). These triple-stained cells were likely hemocytes (possibly plasmatocytes) because they were strongly stained with MitoRed, in contrast to other regions of the epidermis (Figure 2g). These hemocytes were likely stained with BODIPY and Hoechst when they reached the epidermis through the ingestion of cell debris.

**Figure 3:**
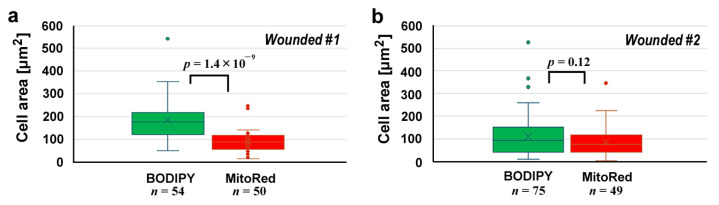
Cell area comparisons at the damage site between BODIPY-positive cells and MitoRed-positive cells after the differential staining protocol. (**a**) The first wounded individual. (**b**) The second wounded individual.

We measured the cell size in each area (Figure 3). In the first wounded individual (Figure 3a), BODIPY-positive cells and MitoRed-positive cells were 184.5 ± 84.3 μm^2^ (*n* = 54) and 92.2 ± 51.7 μm^2^ (*n* = 50), respectively, and their difference was significant (*p* = 1.4 × 10^−9^), suggesting that the BODIPY-positive cells (likely epidermal cells) and MitoRed-positive cells (likely hemocytes) were differentially stained well and mixed at the damage site. However, this was not always the case; in the second wounded individual (Figure 3b), BODIPY-positive cells and MitoRed-positive cells were 112.6 ± 94.9 μm^2^ (*n* = 75) and 88.0 ± 66.9 μm^2^ (*n* = 49), respectively. The difference between them was not statistically significant (*p* = 0.12).

### 3.3. Damage-Induced Cell Migration: First Three Individuals

**Figure 4:**
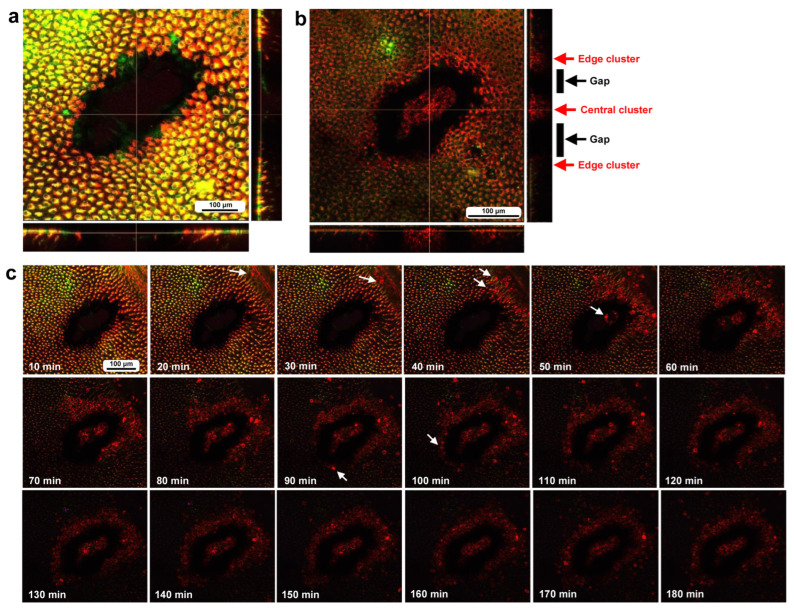
Damage-induced cell migration with a relatively small damage hole (first individual). The tissue was stained with BODIPY and MitoRed via the sandwich method, although the tissue was also treated with Hoechst, which did not work well. The right and bottom rectangular panels associated with the main panel show optical cross sections along the *z*-axis. (**a**) Damage site immediately after the wounding operation. Likely due to overstaining, BODIPY and MitoRed are superimposed, resulting in a yellowish color at this magnification. (**b**) Damage site 3 h postrecording. Cellular clusters are present at the center of the damage site. Cellular clusters are also present around the edge of the damage site. (**c**) Time-lapse images of the damage site for 3 h at 10 min intervals. The time points after the start of time-lapse imaging (the postrecording time; 70 min after the postwounding time) are indicated. The arrows indicate MitoRed-positive migrating hemocytes. See Supplementary Video S4 for further details.

To investigate how wounds were repaired over time at the cellular level, we obtained optical sectional images along the *z*-axis (Figure 4a) before obtaining time-lapse images. The time-lapse imaging started at approximately 70 min postwounding in the first individual. The tissue was stained with MitoRed, BODIPY, and Hoechst via the sandwich method only, although Hoechst staining did not work well. The damage hole was oval with a short diameter of approximately 200 μm, which was considered a relatively small damage hole (Figure 4a). It was devoid of cells at the beginning of imaging (Figure 4a). In contrast, at 3 h postrecording (4 h 10 min postwounding), this damage hole contained a cellular cluster at the center as well as along the edge of the damage area (Figure 4b). Interestingly, an acellular gap remained between the central and edge clusters (Figure 4b). The time-lapse images for 3 h at 10 min intervals clearly revealed long-range cell migration in one direction from the area near the wing vein (lacuna) (upper right) to the central area of damage to cover the center and edge of the damage area (Figure 4c). These migrating MitoRed-positive cells are likely hemocytes, despite the use of only the sandwich method in this case. Cell migration was detected at 20 min postrecording (90 min postwounding) and reached the center of the damage hole at 50 min postrecording (120 min postwounding) (Figure 4c). The center and edge of the damage hole were covered well with migrating cells by 100 min postrecording (170 min postwounding).

**Figure 5:**
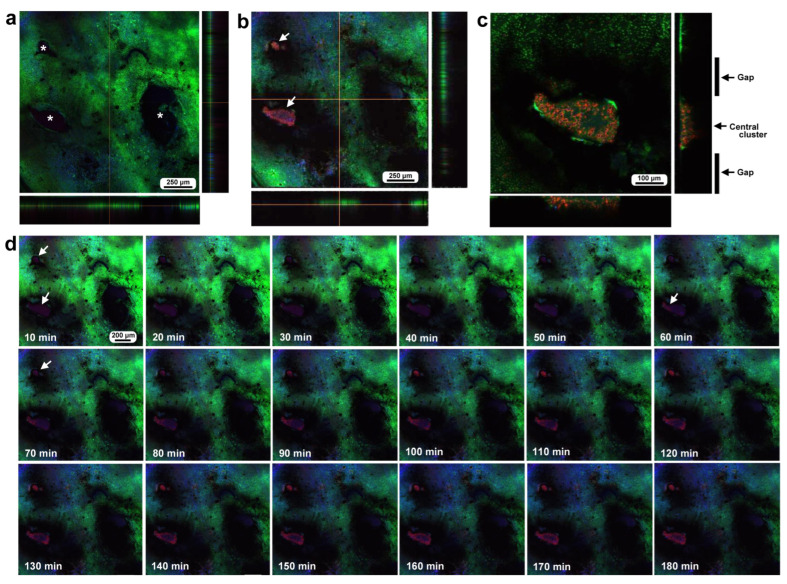
Damage-induced cell migration with three damage holes of various sizes (second individual). The right and bottom rectangular panels associated with the main panel show optical cross sections along the *z*-axis. (**a**) Damage site immediately after the wounding operation. There are three damage holes (asterisks) in this individual. (**b**) Damage site 3 h postrecording. MitoRed-positive cells are observed at the centers of two small damage sites (arrows). (**c**) Magnification of the middle-sized damage hole shown in (**b**). BODIPY signals are observed together with MitoRed signals. (**d**) Time-lapse images of the damage site for 3 h at 10 min intervals. The time points after the start of time-lapse imaging (the postrecording time; 15 min behind the postwounding time) are indicated. The arrows indicate MitoRed-positive cells or areas. See Supplementary Video S5 for further details.

In the second individual, differential staining was performed: the epidermal tissue was stained with BODIPY and Hoechst via the sandwich method, and hemocytes were stained with MitoRed via the injection method. Before time-lapse imaging, we obtained optical cross sections along the *z*-axis. In this individual, three damage holes of different sizes were present (Figure 5a). The largest damage hole was an oval with a short diameter of approximately 300 μm (Figure 5a), which was larger than that of the previous individual (Figure 4). The other two holes (approximately 150 μm and 80 μm in diameter) were smaller than the previous holes. Immediately after the wounding operation, no cells were stained at the damage sites (Figure 5a), but at 3 h postrecording (3 h 15 min postwounding), the centers of the two smaller damage holes were covered with the cells stained with MitoRed (Figure 5b). However, these damage holes appeared to enlarge over time, forming an acellular gap between the central portion and the edge of the damage site, but this could be partly because of the bleaching of BODIPY. A high-magnification image of one of the damage sites also revealed BODIPY-stained cells surrounding the MitoRed-stained cellular cluster (Figure 5c). In this instance, the edge of the damage site did not accompany the cellular cluster (Figure 5c). In contrast, the largest damage holes presented no hemocytes. Time-lapse imaging started approximately 15 min postwounding (135 min postpupation) for 3 h at 10 min intervals, which revealed that the central portions of the small damage holes were covered gradually by MitoRed-positive cells. However, the MitoRed-positive cells seemed to arrive as early as 40 min postrecording (55 min postwounding), although weak MitoRed signals were already observed 10 min postrecording (25 min postwounding) (Figure 5d). The damage hole gradually expanded over time (Figure 5d).

**Figure 6:**
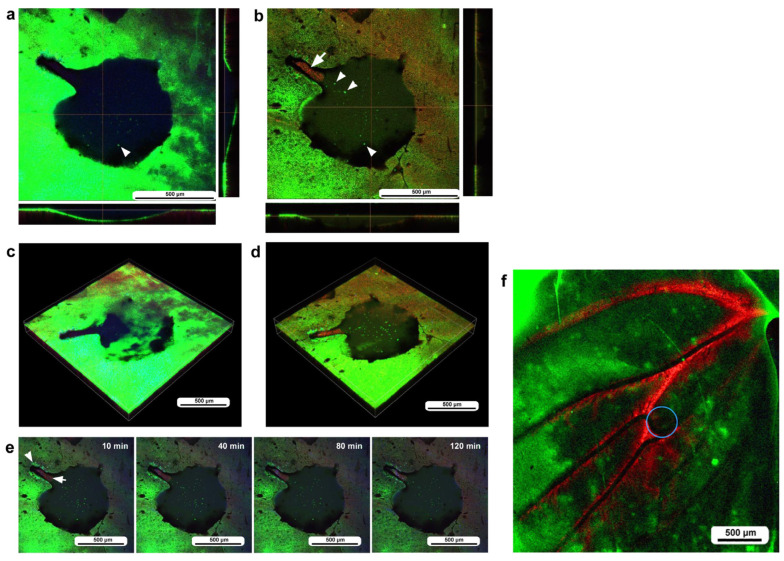
Damage-induced cell migration with a relatively large damage hole but with a cleavage-like portion (third individual). The right and bottom rectangular panels associated with the main panel show optical cross sections along the *z*-axis. (**a**) Damage site immediately after the wounding operation. The optical cross sections at the right and bottom indicate that the epidermis at the damage site was still present and curved, although it was discontinuously broken. There was a narrow cleavage-like portion on the upper left side. The arrowhead possibly indicates a BODIPY-positive epithelial cell. (**b**) Damage site 3 h postrecording. MitoRed-positive cells are observed at the center of the narrow cleavage-like region (arrow), but not in the larger damage area. Arrowheads possibly indicate BODIPY-positive epidermal cells. (**c**) Three-dimensional reconstruction of (**a**). A broken epidermis was clearly observed in the damaged area. (**d**) Three-dimensional reconstruction of (**b**). The broken epidermis was largely cleared, and numerous BODIPY-positive cells were present at the center of the damaged area. (**e**) Time-lapse images of the damage site for 2 h at 10 min intervals. Only representative images are shown. At 10 min, MitoRed-positive hemocytes aggregate at the center of the narrow cleavage-like portion of the damage hole (arrow) with a gap to the edge. The edge of this region may have active epidermal cells (arrowhead). The time points after the start of time-lapse imaging (the postrecording time; 15 min behind the postwounding time) are indicated. See Supplementary Video S6 for further details. (**f**) Low magnification image of a large wing area. The damage site is indicated by a circle.

In the third individual, differential staining was performed as in the previous individual. The damage hole was larger than those of the previous individuals, approaching 700 μm in diameter, but with a narrow cleavage-like portion (approximately 80 μm in width) on the upper left side (Figure 6a). Optical cross sections along the *z*-axis revealed that immediately after the wounding operation, a discontinuous deformed epidermis was still present at the deeper position of the damage hole, but there were no hemocytes at the damage sites (Figure 6a). By the time of 3 h postrecording (195 min postwounding), the center of the cleavage-like portion of the damaged area was covered with hemocytes stained with MitoRed (Figure 6b). In contrast, there were no MitoRed-positive cells in the larger area of the damage hole, although there were a small number of BODIPY-positive cells (Figure 6b), suggesting that migrating hemocytes could not settle at the center of the large hole. The three-dimensional reconstruction of the optical images confirmed these interpretations (Figure 6c,d). BODIPY-positive cells at the center of the large hole could be surviving epidermal cells or newly migrating epidermal cells. Time-lapse imaging started approximately 15 min postwounding, which revealed that MotoRed-positive hemocytes arrived at the cleavage-like damage site within 80 min postrecording (95 min postwounding), although the number of hemocytes seemed to increase over time (Figure 6e). Importantly, the BODIPY-positive epidermal cells around the cleavage-like damage site also moved toward the hole by the end of the recording. Cells migrating along the wing vein were observed approximately 40 min postrecording, although they were not clear in still images.

To understand the role of wing veins (lacunae) in supplying hemocytes to damage sites, we performed the same operation (including fluorescent dyes, staining method, and postwounding time) in a different individual and obtained a low-magnification image of the large wing area (Figure 6f). We noted that MitoRed-positive hemolymph was supplied along the wing vein, possibly from the basal to peripheral areas. Hemocytes likely use this route passively to access the damage site.

### 3.4. Damage-Induced Cell Migration: Two Additional Individuals with FB28

**Figure 7:**
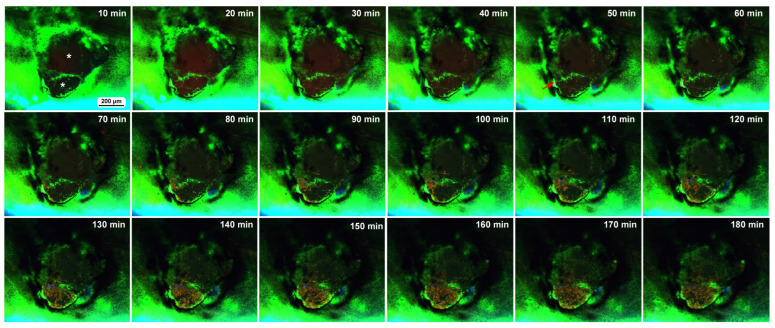
Time-lapse images of damage-induced cell migration with a double-deck damage hole (fourth individual). The time points after the start of time-lapse imaging (the postrecording time; 5 min after the postwounding time) are indicated. Two asterisks in the 10 min image indicate two compartments (holes) of the damage site. Migrating MitoRed-positive cells are observed in the image at 50 min postrecording (red arrow) and afterward. The time points after the start of time-lapse imaging (the postrecording time; 5 min after the postwounding time) are indicated. See Supplementary Video S7 for further details.

We also observed the damage sites of two additional individuals with FB28 (blue), which stains chitin, an important component of the cuticle. Here, BODIPY and FB28 were used in the sandwich method to stain epidermal cells, and MitoRed (red) was used in the injection method to stain hemocytes in the hemolymph. We simply performed time-lapse imaging approximately 5 min postwounding for 3 h at 10 min intervals. The first individual here had a double-deck damage hole of approximately 400 μm in diameter; there was a small compartment (i.e., a small damage hole of approximately 150 μm in short diameter) surrounded by epidermal cells at the bottom of a larger damage hole (Figure 7). FB28-positive objects were observed on the right side of the damage hole but did not seem to have any effect on the cellular dynamics. MitoRed-positive hemocytes were first supplied only to the small damage hole from an entry point in one direction 50 min postrecording (55 min postwounding). Many MitoRed-positive cells subsequently rushed into the hole, and importantly, were mixed with BODIPY-positive cells. By the time of 3 h postrecording, the small hole was completely covered with cells. Simultaneously, the migrating cells also moved to the large hole. BODIPY-positive epidermal cells that constituted the border between the small and large holes seemed to be pushed up by MitoRed-positive cells to spread into the large hole. Interestingly, the damage hole was covered with MitoRed-positive hemocytes and BODIPY-positive epidermal cells, with no clear gaps in this individual.

**Figure 8:**
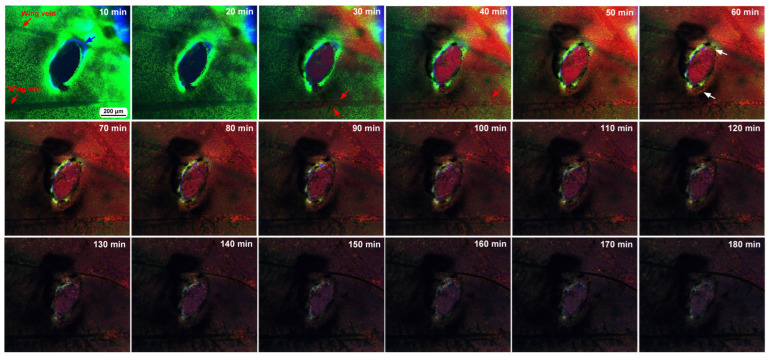
Time-lapse images of damage-induced cell migration with a relatively small damage hole (fifth individual). The time points after the start of time-lapse imaging (the postrecording time; 5 min after the postwounding time) are indicated. Migrating MitoRed-positive cells are observed in the image at 30 min postrecording and afterward (red arrows). There are FB28-positive objects surrounding the edge of the damage hole (blue arrow in the 10 min image). MitoRed-positive cells entering the damage hole are indicated in the image at 60 min (white arrows). The time points after the start of time-lapse imaging (the postrecording time; 5 min after the postwounding time) are indicated. See Supplementary Video S8 for further details.

The second individual was subjected to the same procedure, and time-lapse imaging started at approximately 5 min postwounding. The second individual had an oval damage hole with a short diameter of 200 μm (Figure 8). Importantly, this oval hole was associated with two wing veins. We noticed that the edge of this damage hole was delineated by FB28 signals, suggesting that chitin or cuticle secretion may be quickly executed immediately after damage by epidermal cells or local hemocytes, although the possibility that these FB28 signals are remnants of the pupal cuticle cannot be excluded. We observed MitoRed-positive hemocytes migrating along the anterior and posterior wing veins from the basal region toward the damage site 30–40 min postrecording (35–40 min postwounding). That is, this damage hole was accessed by hemocytes from the anterior and posterior sides simultaneously. By the time of 60 min postrecording (65 min postwounding), MitoRed-positive cells entered the damage hole. BODIPY signals were detected around the edge of the damage hole. In this individual, MitoRed-positive hemocytes in the damage hole were scattered, and the damage hole was not filled. No cellular clusters were formed.

### 3.5. Damage Repair and Chitin Dynamics: Three Additional Individuals with FB28

**Figure 9:**
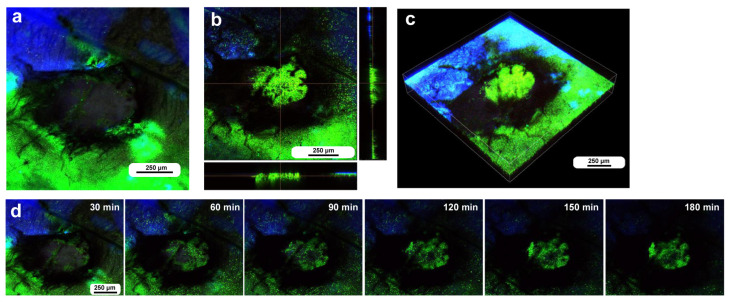
Damage-induced cell migration with a relatively large hole (sixth individual). (**a**) Damage site immediately after the wounding operation. (**b**,**c**) Damage site 3 h postrecording. The right and bottom rectangular panels associated with the main panel show optical cross sections along the *z*-axis. A thick cellular cluster is observed at the center. (**d**) Time-lapse images for 3 h at 10 min intervals. The time points after the start of time-lapse imaging (the postrecording time; 5 min after the postwounding time) are indicated. Only representative images are shown. See Supplementary Video S9 for further details.

We also observed the damage sites in three additional individuals treated with FB28 to understand the cellular and chitin dynamics of the damage site. To do so, we performed differential staining; the epidermal tissue was first stained with BODIPY and FB28 via the sandwich method, and the hemocytes were then stained with MitoRed and SYBR Green I via the injection method, although one of the two dyes (MitoRed or SYBR) did not work well when these two dyes were used simultaneously. In the first individual, immediately after the wounding operation, the damage site was free of cells (Figure 9a). This damage hole was relatively large; its short diameter was approximately 500 μm, but at approximately 3 h postrecording, the central portion of the damage site was covered with cells stained with SYBR (Figure 9b,c), suggesting the migration of hemocytes. Blue FB28 signals were not observed well in the central region (Figure 9b,c). As in previous cases, there was an acellular gap between the central cluster and the edge of the damage hole. However, no cluster formation was observed at the edge, suggesting the priority of the central cluster in the settlement of migrating cells. Time-lapse imaging for 3 h at 10 min intervals started 5 min postwounding and revealed that SYBR-positive cells reached the damage hole by 30 min postrecording (35 min postwounding) (Figure 9d). In this case, we could not reveal the cellular entry points to the damage site.

**Figure 10:**
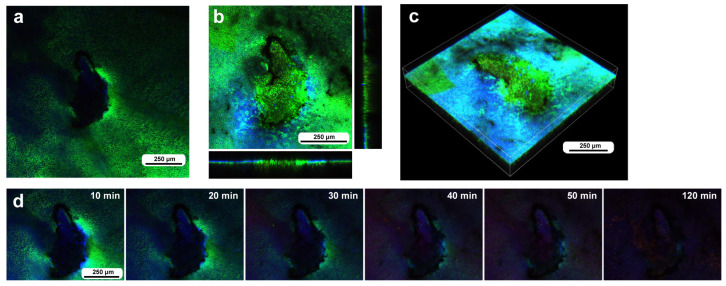
Damage-induced cell migration with a relatively small oval damage hole (seventh individual). (**a**) Damage site immediately after the wounding operation. (**b**,**c**) Damage site 3 h postrecording. The right and bottom rectangular panels associated with the main panel show optical cross sections along the *z*-axis. The damage hole is completely covered with cells. (**d**) Time-lapse images for 3 h at 10 min intervals. The time points after the start of time-lapse imaging (the postrecording time; 5 min after the postwounding time) are indicated. Only representative images are shown. See Supplementary Video S10 for further details.

The second individual subjected to the same procedure and the same timeline had an oval damage hole of approximately 300 μm in short diameter. This damage hole was located on the wing vein near the branching point. No cells were detected in the damage hole at the beginning of the recording (Figure 10a). This damage hole was completely covered with SYBR-positive and/or MitoRed-positive hemocytes without any gap by the time of 3 h postrecording (185 min postwounding) (Figure 10b,c). Unfortunately, because of bleaching, we could not obtain precise time information when these cells reached the damage hole in the time-lapse imaging (Figure 10d), but migrating cells were first supplied from wing veins and then also from regions without wing veins, as if the wing veins were express highways to the damage site. A small number of blue FB28 signals were observed in the central region (Figure 10c), but these FB28 signals may either originate from damaged cuticle remnants or be newly secreted for repair.

**Figure 11:**
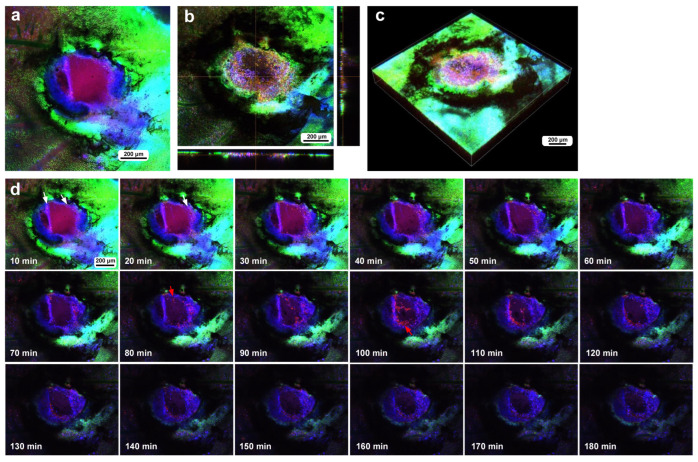
Damage-induced cell migration with FB28-positive objects surrounding the edge of the damage hole (eighth individual). (**a**) Damage site immediately after the wounding operation. (**b**,**c**) Damage site 3 h postrecording. The right and bottom rectangular panels associated with the main panel show optical cross sections along the *z*-axis. The damage hole is completely covered with cells. (**d**) Time-lapse images for 3 h at 10 min intervals. MitoRed-positive cells at 10–20 min (white arrows) and 80–110 min (red arrows) are indicated. The time points after the start of time-lapse imaging (the postrecording time; 5 min after the postwounding time) are indicated. See Supplementary Video S11 for further details.

The third individual, subjected to the same procedure and timeline, had a damage hole 300 μm in diameter, excluding the surrounding FB28-positive regions, or 600 μm, including the FB28-positive regions, immediately after the wounding operation (Figure 11a). This FB28-positive region likely contains damaged cuticle remnants. There were already MitoRed-positive cells along the edges of FB28-positive objects at this point (Figure 11a). Then, at approximately 3 h postrecording, the damage site was covered well with numerous MitoRed cells as well as FB28 signals (Figure 11b,c). In this case, the center of the damage hole had fewer cells than did the peripheral regions. The surrounding gap regions seemed to increase; however, this increase could be due to bleaching. Time-lapse recording for 3 h at 10 min intervals revealed that MitoRed-positive cells were present 10–20 min postrecording (15–25 min postwounding) (Figure 11d). The number of MitoRed-positive cells increased over time until 110 min postrecording (115 min postwounding), and they seemed to move vigorously in the damage site. Importantly, the FB28-positive objects gradually expanded to the center to close the damage hole over time (Figure 11d).

### 3.6. Long-Term Image Recording: Migration of BODIPY-Positive Cells

Thus far, we recorded time-lapse images for 180 min or less, on the assumption that major cell migration events take place within this time span. However, longer recordings may be more informative. Here, we observed the damage sites of two individuals with long-term imaging for 8.3 h and 24 h. Again, differential staining was performed; the epidermal tissue was first stained with BODIPY and Hoechst via the sandwich method, and the hemocytes were then stained with MitoRed via the injection method.

**Figure 12:**
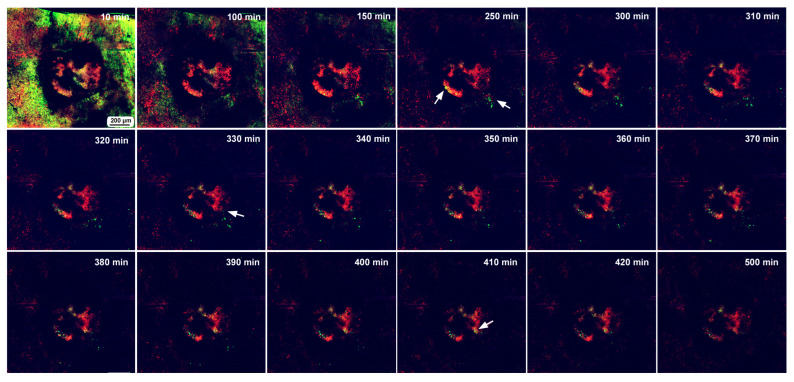
Long-term time-lapse image recording of damage-induced cell migration with a relatively large hole for 500 min at 10 min intervals (ninth individual). The time points after the start of time-lapse imaging (the postrecording time; 3 h after the postwounding time) are indicated. Arrows indicate migrating or clustering BODIPY-positive cells. Only representative images are shown. See Supplementary Video S12 for further details.

In the first individual, image recording started 3 h postwounding and continued for 500 min (8.3 h). This damage hole was relatively large, with a diameter of approximately 650 μm (Figure 12). The center of the damage site was already covered with MitoRed-positive cells at the beginning of the recording (Figure 12), suggesting the migration of hemocytes before 3 h postwounding. An acellular gap region was observed around the central cellular cluster. Rattling movements of BODIPY-positive cells were observed around the time point of 250 min postrecording, and these cells moved toward the central portion of the damage during the period of 330–410 min postrecording.

**Figure 13:**
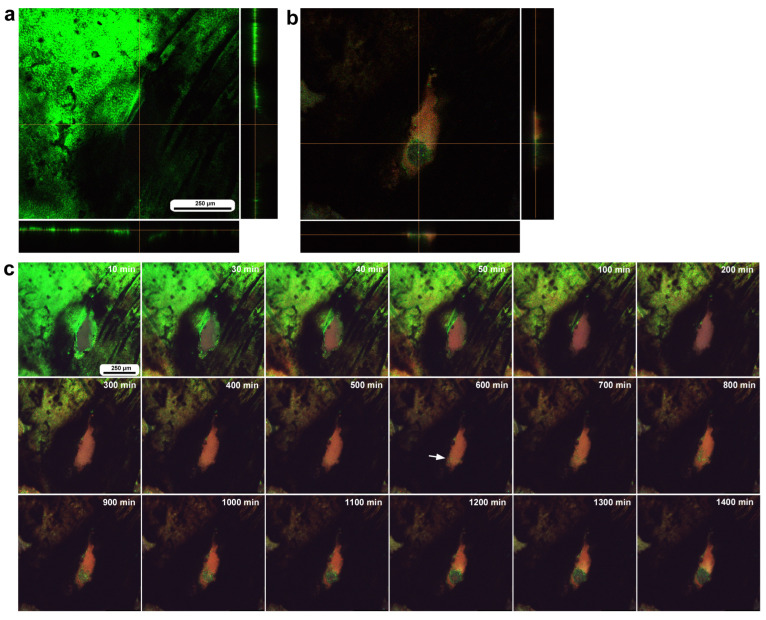
Long-term time-lapse imaging of damage-induced cell migration with a relatively small hole (tenth individual). The right and bottom rectangular panels associated with the main panel in (**a**,**b**) show optical cross sections along the *z*-axis. (**a**) Damage site immediately after the wounding operation. (**b**) Damage site 24 h postrecording. (**c**) Time-lapse images for 24 h at 10 min intervals. The time points after the start of time-lapse imaging (the postrecording time; 30 min after the postwounding time) are indicated. An arrow indicates emerging BODIPY-positive cells. Only representative images are shown. See Supplementary Video S13 for further details.

In the second individual subjected to the same staining procedure, image recording started 30 min postwounding and continued for 1440 min (24 h). This individual had a relatively small oval damage hole approximately 120 μm in short diameter; however, this size was uncertain because of bleaching or incomplete staining around the damage site (Figure 13a). No cells were detected in the damage hole at the beginning of the recording (Figure 13a), but the damage hole was occupied by MitoRed-positive cells as well as BODIPY-positive cells 24 h postrecording (Figure 13b). Time-lapse imaging revealed that MitoRed-positive cells emerged by the time of 30 min postrecording (Figure 13c). BODIPY-positive cells emerged by the time of 600 min postrecording and actively formed a cellular cluster at the center of the damage hole (Figure 13c), suggesting that the migration of epidermal cells was a relatively late event after the migration of hemocytes. The cellular cluster did not completely cover the damage hole within 24 h of image recording, but likely, the cluster was still in the middle of the growth phase at the end of the image recording.

### 3.7. Damage-Induced Calcium Oscillations

**Figure 14:**
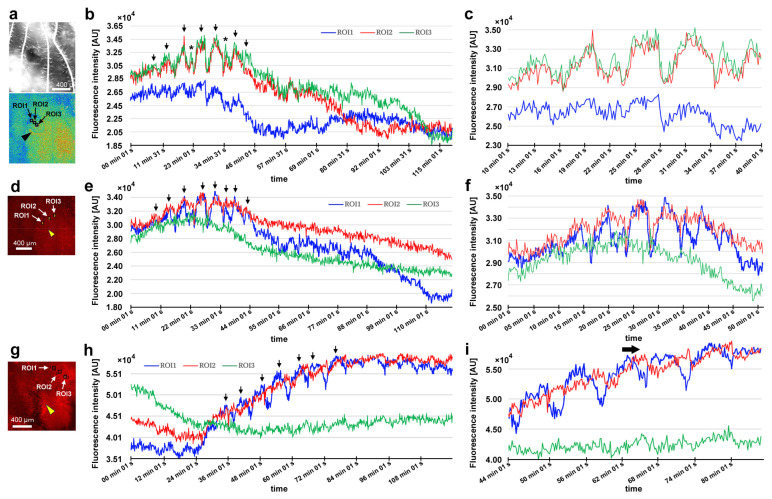
Damage-induced calcium oscillations (first and second individuals). The arrowheads indicate the center of the damage site. (**a**–**c**) Recordings from the first individual for calcium imaging. (**a**) Bright-field image (top) and fluorescence intensity heat-map image of CalBryte (bottom) at the damage site. These modes of visualization were often used to allocate the damage site and decide where to place ROIs. Three ROIs are placed. (**b**) Fluorescence intensity recordings from the three ROIs for 120 min. Arrows indicate calcium oscillation peaks. Asterisks indicate small peaks. (**c**) Detailed fluorescence intensity recordings shown in (**b**) for the period of 10–40 min. (**d**–**f**) Recordings from the same individual with different ROIs. (**d**) Fluorescence intensity image of CalBryte at the damage site. Three ROIs are placed. (**e**) Fluorescent intensity recordings from the three ROIs for 120 min. Arrows indicate calcium oscillation peaks. (**f**) Detailed fluorescence intensity recordings shown in (**e**) during the first 50 min. (**g**–**i**) Recordings from the second individual for calcium imaging. (**g**) Fluorescence intensity image of CalBryte at the damage site. Three ROIs are placed. (**h**) Fluorescence intensity recordings from the three ROIs for 120 min. Arrows indicate the calcium oscillation peaks of ROI1. (**i**) Detailed fluorescence intensity recordings shown in (**h**) for the period of 44–90 min. The horizontal arrow indicates the phase lag between ROI1 and ROI2.

After damage, we recorded the relative changes in calcium signals using CalBryte at and near the damage site. In the first individual, three ROIs were placed sequentially in the central portion of the damaged area (Figure 14a). During the recording period of 2 h, we observed calcium oscillations with peak intervals of approximately 7 min during the period of 10–40 min postrecording from all the ROIs (Figure 14b,c). Calcium oscillations in these ROIs appeared to be largely synchronized, but ROI3 peaks seemed to be the earliest, followed by ROI2 and ROI1. The signal intensity also largely followed this order. That is, the calcium waves likely propagated with spatial attenuation within a limited distance from the center to the periphery of the damage site. In the same individual, three new ROIs were set: ROI1 was placed at the center of the damage site, ROI2 was placed at the edge of the damage site, and ROI3 was placed at a small distance from the edge (Figure 14d). Again, we observed calcium oscillations at 5–7 min intervals in ROI1 and ROI2; they were largely synchronized, but ROI3 did not show notable oscillations (Figure 14e,f), suggesting that calcium waves propagated locally within a few hundred micrometers. Interestingly, after the major oscillations during the period of 10–40 min postrecording faded, small oscillations reappeared during the period of 55–90 min postrecording only in ROI1 but not in ROI2 or ROI3, suggesting that the central region of the damage site may be more responsible for calcium oscillations and the propagation of calcium waves.

**Figure 15:**
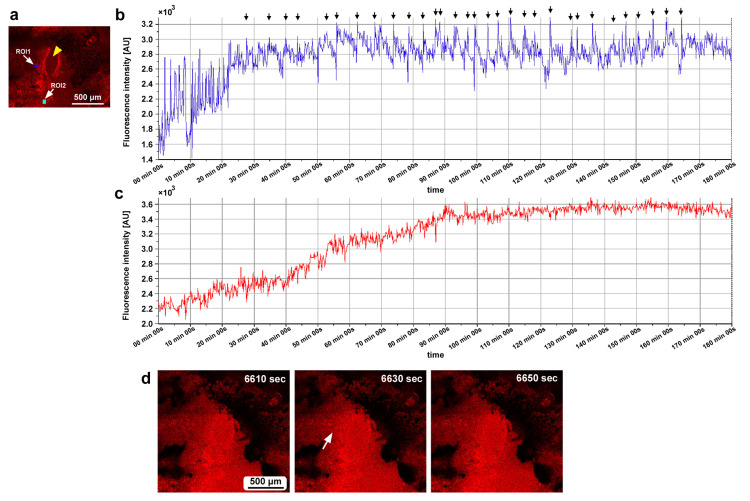
Damage-induced calcium oscillations (third individual for calcium imaging). (**a**) Fluorescence intensity image of CalBryte at the damage site. The yellow arrowhead indicates the center of the damage site. Two ROIs are placed. (**b**) Fluorescence intensity recordings from ROI1 for 180 min. Arrows indicate calcium oscillation peaks. (**c**) Fluorescence intensity recordings from ROI2 for 180 min. (**d**) Representative time-lapse images of CalBryte fluorescence intensity for 180 min. The time points after the start of time-lapse imaging (the postrecording time) are indicated. The arrow indicates the region of an expanding calcium wave (an increase in fluorescence intensity) at 6630 s. Only representative images are shown. See Supplementary Video S14 for further details.

In the second individual, three ROIs were placed at a small distance from the damage site (Figure 14g). We observed calcium oscillations with 7 min intervals in the period of 25–80 min postrecording in ROI1 and ROI2 (Figure 14h,i). A phase lag was observed between ROI1 and ROI2. ROI1 peaks were earlier than those of ROI2, although ROI1 was placed at a position farther than ROI2 from the center of the damage site, suggesting a complex mechanism of calcium oscillations and wave propagation. ROI3 did not exhibit clear oscillations. In the third individual, two ROIs were placed (Figure 15a), and various types of oscillations continued for the entire recording period (180 min) in ROI1 (Figure 15b). For the first 20 min postrecording, calcium spikes of approximately 1 min intervals were recorded, after which oscillations with 5–7 min intervals were observed (Figure 15b). In contrast, ROI2 did not exhibit any clear oscillations or spikes (Figure 15c). Time-lapse images revealed visually recognizable calcium waves expanding from the damage hole to the surroundings (Figure 15d).

**Figure 16:**
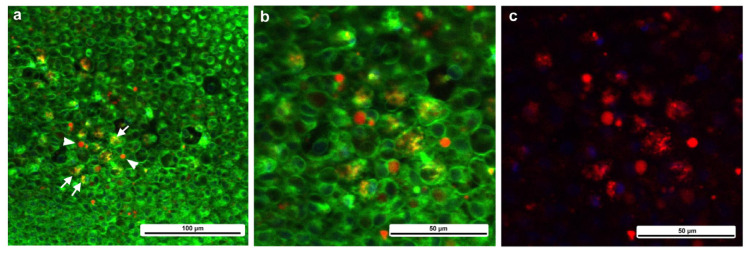
CalBryte-positive signals at the damage site. The tissue was stained with BODIPY and CalBryte. (**a**) CalBryte-positive red signals. Some of them are yellow due to colocalization with BODIPY-positive green signals. The arrows indicate diffuse colocalization of CalBryte with BODIPY. Arrowheads indicate concentrated CalBryte red signals enclosed by a BODIPY-positive membranous structure. (**b**) Magnification of (**a**). (**c**) Same visual field as (**b**), but with only red signals.

Two types of CalBryte-positive red signals were observed at the damage site (Figure 16). One was diffusely colocalized with BODIPY, which is an intracellular membrane-associated structure, likely the endoplasmic reticulum, and the other showed globular red signals surrounded by BODIPY-positive membranous structures (Figure 16). These CalBryte-positve globules were approximately 5 μm or less in diameter. The former is likely the origin of ER-associated calcium oscillations, considering that CalBryte is thought to accumulate in the cytoplasm. The latter is uncertain; however, these relatively large globules may represent calcium stores.

### 3.8. Ruthenium Red Affected Damage-Induced Calcium Oscillations

**Figure 17:**
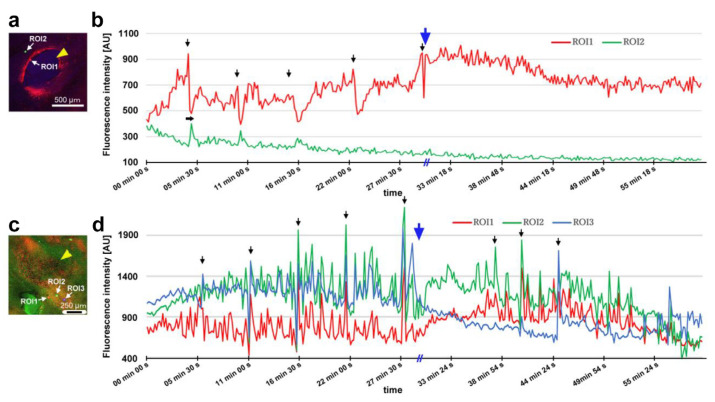
Ruthenium red injection during calcium oscillations after damage. The arrowheads indicate the damage sites. The small black arrows indicate the calcium oscillation peaks. The large blue arrows indicate the time points of ruthenium red injection. (**a**,**b**) Recordings from the fourth individual for calcium imaging. (**a**) Fluorescence intensity image of damage site. Two ROIs are placed. (**b**) Fluorescence intensity recordings from the two ROIs for 60 min. The horizontal arrow indicates the phase lag between ROI1 and ROI2. (**c**,**d**) Recordings from the fifth individual for calcium imaging. (**c**) Fluorescence intensity image of the damage site. Three ROIs are placed. (**d**) Fluorescence intensity recordings from three ROIs for 60 min.

While recording calcium signals after the wounding operation, we administered ruthenium red, a general inhibitor of calcium signals, to examine whether calcium oscillations were sensitive to this treatment. In the first individual, two ROIs were placed at the edge of the damage hole (Figure 17a). Calcium oscillations were observed at 5 min intervals before the injection of ruthenium red, but the oscillations from ROI1 occurred first, followed by those from ROI2, with much smaller amplitudes (Figure 17b), suggesting attenuated calcium wave propagation from the very edge of the damage site to the peripherals. ROI1 oscillations were disrupted by the injection of ruthenium red at the 30 min time point without clear reappearance of oscillations afterward (Figure 17b). In the second individual, three ROIs were placed (Figure 17c). We observed calcium spikes for approximately 1 min before the injection of ruthenium red (Figure 17d). Among these spikes, those with high amplitudes were observed at 5 min intervals. These spikes were also disrupted temporarily by the injection of ruthenium red, but the spikes restarted within 10 min, although with smaller amplitudes.

### 3.9. Ruthenium Red Affected Color and Scale Development

**Figure 18:**
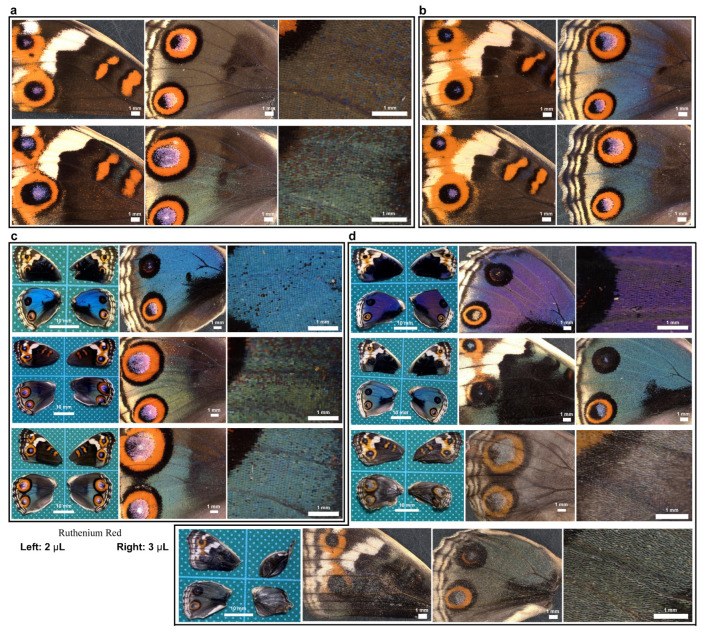
Effects of ruthenium red injection on color patterns and scale development. The panels in a single row represent images from the same individual. (**a**) Untreated individuals from the sibling population for a 2 μL injection. (**b**) Untreated individuals from the sibling population for a 3 μL injection. (**c**) Treated individuals with a 2 μL injection (*n* = 3). (**d**) Treated individuals with a 3 μL injection (*n* = 4).

Here, we tested the involvement of calcium signals in wing development by injecting ruthenium red into pupae and examining adult wings after eclosion. Untreated sibling individuals were used as controls (Figure 18a,b). Individuals with ruthenium red injection (2 μL) (*n* = 3) appeared to have fragile and wrinkled wings and relatively large anterior eyespots in the dorsal hindwings in both males and females (Figure 18c), although it was not certain whether this eyespot enlargement was caused by ruthenium red injection because there are natural individual variations in eyespots in this species. However, all individuals with a larger ruthenium red injection volume (3 μL) (*n* = 4) presented the following aberrant features (Figure 18d). One male individual had an unusual purple color instead of a blue color (Figure 18d, top). Another male individual was pale blue (Figure 18d, second from the top), suggesting that cuticle structures on a scale (either cover or ground scale) were not well developed. Two females showed unsuccessful eclosion with wrinkled right wings, and their wing color patterns were very pale with low contrast (Figure 18d, third and fourth from the top). High-magnification images suggested that the scales were not well developed, and their scales were pale (Figure 18d, bottom two). Most of the treated individuals appeared to have large anterior eyespots in the dorsal hindwings. These results suggest that calcium signals are involved in scale development, including the development of structural and pigment colors, and may also be involved in color pattern formation, such as eyespot size.

### 3.10. Repair Was Not Sensitive to Ruthenium Red Injection

**Figure 19:**
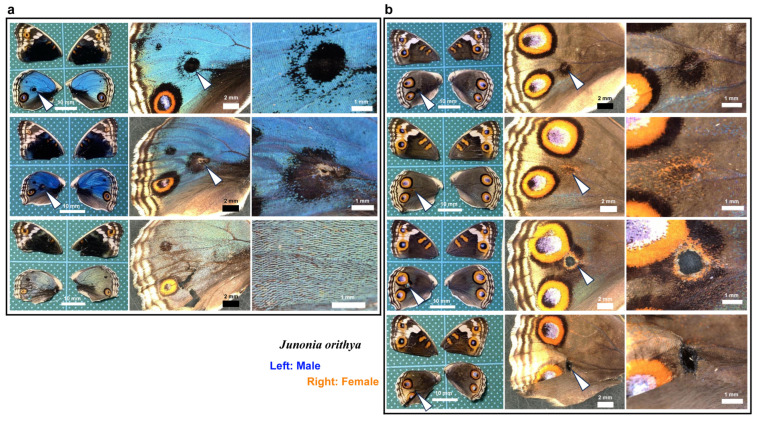
Wing repair and ectopic color patterns after simultaneous puncture damage and ruthenium red injection. The panels in a single row represent images from the same individual. The arrowheads indicate the damage sites and the damage-induced ectopic color patterns. (**a**) Male individuals. (**b**) Female individuals. The bottom two individuals have a hole (seen as a black area) at the center of damage.

Here, we tested whether calcium signals might also play an important role in wound healing and ectopic color pattern induction via simultaneous puncture damage and ruthenium red injection. In the first male individual (Figure 19a, top), the damage-induced black spot had an outer ring similar to an eyespot. In the second male individual (Figure 19a, middle), the center of the damage-induced black spot lacked scales. The black spot of the second individual with a scar was larger than that of the first individual without a scar, suggesting that the induced black spot size was proportional to the severity of damage. These black spots were similar to those observed in the absence of ruthenium red (Figure 1a). Importantly, in one male individual, the damage seemed to be largely repaired despite the scale size and color being affected by ruthenium red (Figure 19a, bottom). In females, black spots were induced, as in males (Figure 19b, top), but they were often associated with orange rings (Figure 19b, second from the top). In one female individual, a large hole remained, and orange and black rings were present around the hole, similar to an eyespot, which fused with normal eyespots (Figure 19b, third). This result is similar to that observed in the absence of ruthenium red (Figure 1b). One individual presented a wing hole without color pattern induction (Figure 19b, fourth). Among the treated individuals (*n* = 25), five presented with a scar or hole at the center of the damage. These results suggest that even with ruthenium red, the damage was repaired normally, and ectopic color patterns were induced “normally” in most individuals.

Here, we assume that the presence of a scar or hole is an indicator of repair inefficiency. When ruthenium red was injected after the wounding operation, five individuals presented with a scar or hole at the damage site. On the other hand, when no ruthenium red injection was performed after the wounding operation, four individuals presented with a scar or hole at the damage site (see Section 3.1). Between the two groups with or without ruthenium red injection, there was no statistically significant difference in terms of the presence of a scar or hole at the damage site (*p* = 0.72; Fisher’s exact test), suggesting that the repair system itself was not sensitive to the temporary inhibition of calcium signals after damage. Similarly, ectopic color patterns are unlikely to be sensitive to the temporary inhibition of calcium signals because we obtained many individuals with ectopic color patterns after the injection of ruthenium red, although their patterns varied.

## 4. Discussion

**Figure 20:**
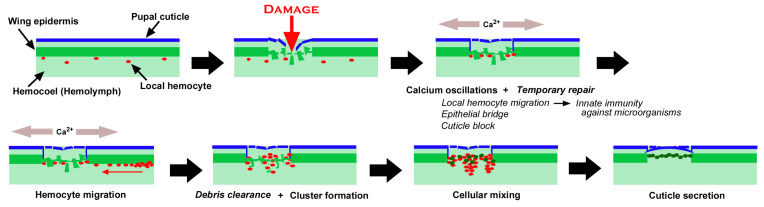
Summary of the present study on wound healing in butterfly pupal wing tissue. The speculative mechanisms not observed in this study are shown in italics. Mechanical damage induces calcium oscillations at the damage site. Simultaneously, the temporary repair system is activated, which includes local hemocyte migration, epithelial bridges, and cuticle blocks. In response to calcium oscillations, hemocytes migrate to the damage site from the nearest wing vein. Hemocytes may clear cellular debris and form clusters. Some epidermal cells around the damage site move to the damage center and edge to form a cellular mixture with hemocytes. The epidermal cells then reconstruct the epidermal sheet and the pupal cuticle to seal the damage site.

We studied wound healing and the associated ectopic color patterns of butterfly wings, focusing on the cellular dynamics and calcium oscillations at the damage site by bioimaging techniques. Because we performed the puncture damage manually, we could not avoid the variability of the damage site in size and shape. This operational variability was probably reflected in the various levels of wing repair and ectopic color patterns among the treated individuals. It is likely that an individual with severe damage formed a large ectopic eyespot-like color pattern around an unsealed damage hole in adult wings, and that an individual with light damage formed a small ectopic color pattern (or no color pattern) without a scar or hole at the damage site in adult wings. Nevertheless, in most individuals that eclosed after the wounding operation, the damage was repaired completely with minor ectopic color patterns in adult wings in our experimental system, indicating that the wound-healing process was robust and normally executed in the treated individuals in our experimental system. Additionally, in terms of methodology, our staining procedures, including the differential staining method, were not perfect. We often observed that one of the two fluorescent dyes used simultaneously did not work well, and the sandwich method stained the hemocytes to various degrees. MitoRed-positive cells (i.e., hemocytes) and BODIPY-positive cells (i.e., epidermal cells) were successfully differentiated in size in the healing damage sites, as shown in Figure 3a, but not in Figure 3b. Nonetheless, we believe that our methodology was acceptable with careful interpretation, and that our differential staining method can be used for rough distinction between epidermal cells in the epidermis and hemocytes in the hemolymph. We did not intend to differentiate hemocyte subtypes in this study, but future studies may use some fluorescent dyes specific to subtypes of hemocytes. Bearing these points in mind, some of the findings of this study are illustrated in Figure 20.

As shown in Figure 20, we speculate the following time course of wound healing in butterfly pupal wing tissues. Before damage, an epidermal sheet is present in association with the pupal cuticle, and local hemocytes are present in the hemocoel [83,84]. Puncture damage breaks the pupal cuticle and underlying epidermis. Many epidermal cells nonetheless seem to be alive after damage, possibly contributing to temporary repair, including epithelial bridge formation (horizontal extension and connection among damaged but surviving epidermal cells in the damage hole) and cuticle block (secretion of the cuticle to temporarily seal the edge of the damaged tissue) around the edge of the damaged tissue, although this temporary repair process is speculative. At the same time, these epithelial cells emit calcium signals, which probably induce taxis signals such as hydrogen peroxide, although this is also beyond the scope of the present study. Local hemocytes in the hemocoel are quickly attracted to the damage site to execute phagocytic functions against invading microorganisms through Toll-like receptors (TLRs), together with the clearance of cell debris. In response to taxis signals that may be induced by calcium signals, additional hemocytes migrate mostly along the nearest wing veins (lacunae) to the damage site, as shown in the present study. These hemocytes may also clear cell debris but form cellular clusters together with surviving epidermal cells. Importantly, novel epidermal cells are then recruited to the damage site, further forming clusters. Epidermal cells secrete cuticle to repair the damaged cuticle, and the damage hole is sealed with epidermal cells, as shown in Figure 10 and Figure 11. This scenario is largely based on the experimental results presented in this study, but includes our speculations, which should be verified or rejected in the future. The migrating cells via the wing veins in a long range in response to damage are likely plasmatocytes (and/or granulocytes) that can phagocytose cell debris and invading microorganisms [4].

The cell migration after damage observed in the present study was notable at the following five points. First, cell migration occurred mainly in defined directions from the nearest wing vein. This is somewhat surprising, considering that the taxis signals may be released randomly in all directions. Indeed, some cells seem to have arrived later at the damage site from different directions. Thus, wing veins may function as express highways for cell migration. In addition, cell migration has a long range, and the migration distance may be much greater than several hundred micrometers (Figure 4 and Figure 8). However, the fast migration of hemocytes along the wing veins is likely passive, because hemocytes are just circulating within the hemocoel. Once circulating hemocytes recognize a chemotactic signal from the damage site when they approach that site passively, they will probably leave the wing vein to travel to the damage site.

Second, migrating cells first arrived at the damage site as early as 15 min postwounding (63 min on average; *n* = 7) on the basis of time-lapse images, with the following arrival times: 120 min (Figure 4), 55 min (Figure 5), 95 min (Figure 6), 55 min (Figure 7), 65 min (Figure 8), 35 min (Figure 9), and 15 min (Figure 11). Early-arrival and late-arrival cells may be composed of different populations of hemocytes, and early-arrival hemocytes may be difficult to record; local hemocytes might have arrived even before that time to eradicate invading microorganisms. In insects, most bacteria (99.5%) invading the hemocoel are eliminated within 1 h of invasion [110].

Third, migrating cells settled at the center of the damage site or at the edge of the damage site, where they formed cellular clusters. The cellular clusters are reminiscent of the regenerative blastema observed in other regeneration systems [9,33,111,112]. Compared with the normal epidermis, the cellular clusters at the damage site seem to be composed of relatively large cells, and they were irregular in shape. The priority of the settlement position for migrating hemocytes seems to be given to either the center or the edge, probably depending on the presence of damaged cells releasing calcium signals or taxis signals.

Fourth, oddly enough, there was an acellular gap between the central and edge clusters in many cases. Alternatively, the damage hole was covered with cells without a gap. In our imaging system, wound healing did not proceed beyond this stage in some individuals and in that case, the physical gap between the central and edge clusters remained. We believe that this gap will eventually be covered by epidermal cells under normal conditions. If so, the damage hole is repaired not only from the edge but also from the central portion simultaneously. In many cases, the damage hole expanded over time, probably partly because of cell death at the edge of the damaged tissue and partly because of bleaching of the fluorescent dyes. In the case of the former, the repair process did not catch up with tissue degradation. However, in some other cases, the damage hole was completely covered with cells. A new cuticle seemed to be added to close the damage hole, as shown in Figure 10 and Figure 11.

**Figure 21:**
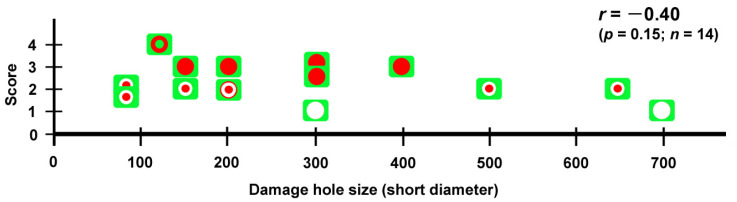
Summary of four repair states (full coverage with epidermal central coverage, full coverage, central coverage, and no coverage) in relation to the size of the damage hole (short diameter). No coverage is shown as a blank circle on a green background. The central coverage is shown as a small red circle at the center of the blank circle. The central coverage may or may not accompany the edge cluster. Full coverage is shown as a large red circle filling the blank circle. Full coverage with epidermal central coverage is shown as a green dot at the center of the red circle. The repair state of the fifth individual (Figure 8) was considered full coverage as the healing process was still ongoing at the end of the recording. Pearson correlation coefficient (*r*), its associated *p*-value (*p*), and the number of damage holes examined (*n*) are shown.

Fifth, not all damage holes attracted hemocytes. In an individual with three damage holes nearby, two were covered by cells, but the largest one was not covered at all (Figure 5). Overall, in our system, there were four states of wing repair: full coverage with epidermal central coverage (as shown in Figure 13), full coverage, central coverage, and no coverage. The four states of wing repair were schematically drawn in relation to the short diameter of the damage hole (Figure 21). It appears that small holes of 200 μm or less can attract hemocytes efficiently and be sealed by cells completely or partially, but large holes of 300 μm or more cannot always do so. When scores were given to the four states of wing repair, they were correlated with the size of the damage hole (Pearson correlation coefficient *r* = −0.40; *n* = 14), although it was not statistically significant (*p* = 0.15). It is likely that the larger damage holes were repaired less efficiently than the smaller holes. This interpretation is consistent with the interpretation of the results for the adult wings shown in Figure 1. Especially, the large damage holes without cellular coverage are consistent with the fact that we obtained adult wings with a large unsealed damage hole at the damage site (Figure 1 and Figure 19). Similarly, we speculate that the three consequences of wound healing in adult wings (i.e., sealed, scar, and hole) partially depend on the physical size of the damage hole but also on the availability of live epidermal cells that release calcium or other signals in the damage hole, which were not always visualized well in this study but likely existed in many individuals with complete coverage. The proliferation of epidermal cells may occur to cover the entire damaged area. However, we did not observe phenoloxidase-mediated melaninization of the damage site by hemocytes. We do not know whether this process is related to butterfly color pattern formation.

In the *Drosophila* wing imaginal disc, wound healing of an ablated portion has been studied intensively, in which an ablated portion is filled from the angular portion of the damage where two edges can meet within a small distance [12], which may be analogous to the cellular coverage from the edge to the center of the damage in the present study. The central coverage with an isolated cellular cluster observed in the present study may be unique to the wound-healing process of a puncture hole. In the *Drosophila* system, filopodia, actin cables, and proliferation of epidermal cells are known to be important [12], and will be examined in the future in our butterfly system. However, the contributions of hemocytes to the wound-healing process may not be well discussed in the *Drosophila* system. It would be interesting to observe the potential effects of hemocyte depletion on the wound-healing process in the future.

From the center and edge of the damage, we observed calcium oscillations, which were observed almost immediately after the wounding operation, likely before the beginning of cell migration. Thus, the release of calcium signals from the damaged epidermis may be the very early step of wound healing. Considering that calcium signals in butterfly pupal wings are relatively slow in propagation (1–10 μm/s) but travel long distances (more than 1 mm), according to a previous study [52], damage-induced calcium signals may be suitable for attracting hemocytes quickly from distant sources. In *Drosophila* pupal wing tissues, calcium signals are considered master regulators of wound healing [20]. This may also be true for butterfly pupal wing tissues. However, calcium signals are not able to attract hemocytes directly because the expansion of calcium waves is likely mediated by IP_3_ via gap junctions [21,22]. To attract hemocytes, signals must be mediated by hemolymph. In this sense, calcium signals may induce the release of other signaling molecules, such as hydrogen peroxide, to attract hemocytes [27]. However, calcium signals may directly mediate the migration of epidermal cells to the damage site. The migration of epidermal cells occurred later than that of hemocytes in the present study. In this event, which may be similar to the epithelial-mesenchymal transition (EMT), epidermal cells must change their shape and size; relatively flat and large cells were observed at the damage sites in the present study, as shown in Figure 2. Under normal conditions without damage, epidermal cells in butterfly pupal wing tissues at this stage are vertically elongated, as revealed by real-time confocal microscopy [84,85] and transmission electron microscopy (TEM) [109]. The formation of cellular clusters is likely an important step in the reconstruction of a new piece of epidermal tissue.

In the present study, calcium oscillations had peak intervals of approximately 5–7 min, but we also observed sharper oscillations with short intervals of 1–2 min, which may be called calcium spikes. Adjacent regions at or around the damage site appeared to exhibit largely synchronized oscillations. However, the central regions of the damage site had slightly higher amplitudes and earlier peaks in most cases, suggesting that oscillations can propagate as calcium waves from the center of the damage site. Moreover, calcium oscillations were not observed in ROIs a few hundred micrometers away from other active ROIs, suggesting that damage-induced calcium waves attenuate over distance and that the range of calcium wave propagation is limited, probably depending on the size of the damage hole, although this propagation range may still be considered a long range in comparison with the cell size. In our analyses, ROIs near the damage site most often had higher intensity changes than ROIs distant from the damage site, but this was not always the case, as shown in Figure 14h. This is probably because some cells near the damage site could not produce calcium oscillations due to severe damage. We did not record more than 180 min, but calcium oscillations seem to mostly cease within this time span, although this may be because of technical reasons such as bleaching of CalBryte and death of the damaged cells of the wing tissue.

Calcium oscillations might have originated from a cytoplasmic region near the ER membranes because CalBryte signals were colocalized with the BODIPY signal, an indication of intracellular membranous structures near the nucleus. However, the contribution of calcium ions from other sources to calcium oscillations cannot be ruled out. Moreover, we observed relatively large CalBryte-positive membrane-enclosed globules. These globular structures were highly positive for CalBryte, suggesting that they may function as calcium stores. These globules may have been previously reported as endosome-like structures but were mostly insensitive to various fluorescent dyes [84].

Considering that calcium signals may function to attract epidermal cells directly and hemocytes indirectly during wound healing, we speculate that during the normal development of butterfly wing color patterns, calcium signals from the eyespot organizer may initiate the migration of surrounding epidermal cells to the prospective eyespot focus to further augment the eyespot organizer. However, calcium signals seem to have multiple functions in butterfly wing development. To understand the physiological function of calcium oscillations, we used ruthenium red, which is known to inhibit various types of calcium transporters and channels, including ER Ca^2+^-ATPase (SERCA), ryanodine receptors, mitochondrial Ca^2+^ uniporters, and TRP and Piezo mechanoreceptors [113,114,115,116,117,118,119]. As expected from a previous study [52], ruthenium red disrupted calcium oscillations in the present study, although the possibility of dislocation of the object cannot be excluded completely. The effect of ruthenium red seems to be transient; smaller spikes restarted within approximately 10 min after treatment in one individual (Figure 17c,d). This transient effect may explain why ruthenium red treatment did not affect healing efficiency under our experimental conditions. Similarly, ectopic color pattern induction by damage was not affected by the injection of ruthenium red, although we obtained one individual with an unsealed damage hole without ectopic color patterns after the injection of ruthenium red, in which ruthenium red might have suppressed the development of ectopic color patterns.

On the other hand, ruthenium red impaired normal scale development in terms of structural and pigment colors and potentially enlarged eyespots in size, suggesting that calcium signals play important roles in various aspects of scale development and color pattern formation in the normal development of butterfly wings. Interestingly, structural colors in butterfly wing scales are produced by the microarchitecture of actomyosin and chitin complexes [120,121]. Calcium is a well-known regulator of actomyosin contraction and relaxation in the muscles [122,123]. If this is applicable to butterfly wings, it is conceivable that in butterfly wings, calcium signals drive the actomyosin complex for normal scale development. However, this potential function of calcium in scale development must be executed a few days postpupation when scales are produced. Thus, aberrant phenotypes induced by ruthenium red may be unrelated to the calcium signals observed during the wound-healing process in this study. Nonetheless, we believe that calcium signals in wound healing drive actomyosin complexes to mechanically close the wound.

Actomyosin-driven mechanical forces play important roles not only in wound healing in *Drosophila* [10,14,15,16,17] but also in the development of many other biological systems. During morphogenesis, the developing epithelium is under compression, which causes mechanical buckling of the epithelium, as shown in the suspended epithelia of the canine kidney [124]. Buckling forces in two-dimensional epithelium may be employed in morphogenesis to produce folds and other complex structures in many developmental systems, including the brain, intestine, and lung [125]. In an organotypic culture of mouse embryonic submandibular glands, buckling forces are provided by the actomyosin complex, which then promotes branching of this epithelial organ [126]. According to a simulation study, an actomyosin ring is likely responsible for epithelial folding [127]. Gastrulation is initiated by a propagating furrow, which is initiated by epithelial buckling in *Drosophila* embryos [128]. Importantly, to drive this buckling, embryo-scale force balance is necessary and sufficient [128]. Similar to mechanical force, calcium waves promote apical extrusion of cells from the epithelium in cultured cells and zebrafish embryos [129,130]. In these systems, IP_3_ receptors and gap junctions are involved in calcium wave propagation [129], similar to the *Drosophila* wound-healing system [21,22,23].

Calcium signals are likely used in normal color pattern formation in butterflies because spontaneous calcium waves are released from the center of the prospective eyespot (i.e., the eyespot organizer) [52]. At the prospective eyespot focus, eyespot organizing cells form a cellular cluster underneath the pupal cuticle spot, which physically distorts the wing epidermis [69,70,71]. This physical distortion may be an initiator of calcium signals from the eyespot organizer and vice versa, which is called the distortion hypothesis [64]. Theoretically, physical distortions can include epithelial thickening, buckling, furrows, and waves driven by actomyosin or other cytoskeletal filaments, which are reminiscent of the differentiation waves proposed by Gordon [131,132,133,134]. Evolutionarily, this distortion mechanism may be considered a co-option of wound-healing mechanisms, and damage-induced ectopic color patterns in butterflies may be considered a consequence of this evolutionary co-option [97]. The fact that calcium signals are a driver of the actomyosin complex might have made it easier for butterflies to evolve structural color in scales because scales require the actomyosin complex. Similarly, this calcium–actomyosin relationship might have made it easier for butterflies to evolve complex color patterns that require the actomyosin complex for physical distortions. Interestingly, thapsigargin-treated *Junonia almana* individuals presented a “reversed-type” color pattern modification, which tends to enlarge eyespots [135], and thapsigargin-treated *Vanessa cardui* can recapitulate its sister species, *Vanessa kershawi*, in terms of wing color patterns [136]. These studies suggest that calcium signals can be employed to determine and diversify wing color patterns across species.

## Data Availability

The original contributions presented in this study are included in the article. Further inquiries can be directed to the corresponding author. Original microscopy images used for figures in this paper can be downloaded at https://zenodo.org/record/14710458 (accessed on 21 January 2025).

## Supplementary Materials

The following supporting information can be downloaded at https://zenodo.org/record/14709810 (accessed on 21 January 2025): Video S1: Three-dimensional reconstruction of a nondamaged site (Figure 2b); Video S2: Three-dimensional reconstruction of a damage site (Figure 2d); Video S3: Three-dimensional reconstruction of another damage site (Figure 2f); Video S4: Damage-induced cell migration (Figure 4); Video S5: Damage-induced cell migration (Figure 5); Video S6: Damage-induced cell migration (Figure 6); Video S7: Damage-induced cell migration (Figure 7); Video S8: Damage-induced cell migration (Figure 8); Video S9: Damage-induced cell migration (Figure 9); Video S10: Damage-induced cell migration (Figure 10); Video S11: Damage-induced cell migration (Figure 11). Video S12: Damage-induced cell migration (Figure 12). Video S13: Damage-induced cell migration (Figure 13). Video S14: Calcium dynamics at the damage site (Figure 15).
